# Vitreomacular traction quantitative cutoffs for the assessment of resolution after ocriplasmin intravitreal treatment

**DOI:** 10.1038/s41598-020-74472-4

**Published:** 2020-10-16

**Authors:** Alessandro Arrigo, Alessandro Calamuneri, Alessandro Bordato, Emanuela Aragona, Luisa Pierro, Francesco Bandello, Maurizio Battaglia Parodi

**Affiliations:** 1 Department of Ophthalmology, IRCCS Ospedale San Raffaele, University Vita-Salute, Via Olgettina 60, 20132 Milan, Italy; 2 IRCCS Centro Bonino-Pulejo Neurolesi, Messina, Italy

**Keywords:** Neuroscience, Biomarkers, Health care, Medical research, Risk factors

## Abstract

This study aimed to assess optical coherence tomography (OCT) parameters associated with vitreomacular traction (VMT) resolution after ocriplasmin intravitreal injection and also associated with the development of vitreomacular complications. Study designed was a retrospective case series. Structural OCT images were acquired at baseline and over the follow-up after treatment. We developed a mathematical model to provide quantitative parameters associated with VMT resolution. Moreover, we adopted the same model to assess the quantitative parameters associated with development of further vitreomacular complications or with the worsening of the coexisting condition. Main outcome measures were BCVA, central macular thickness (CMT), VMT reflectivity, VMT size, VMT resolution, epiretinal membrane (ERM), macular holes. 73 eyes of 73 VMT patients (mean age 73 ± 9 years) were recruited. The mean follow-up duration was 2.6 ± 1.1 years. Mean baseline BCVA was 0.38 ± 0.18 LogMAR, improving to 0.26 ± 0.20 at the end of the follow-up (*p* < 0.01). Baseline CMT was 431 ± 118 µm, improving to 393 ± 122 µm at the end of the follow-up (*p* < 0.01). 38/73 eyes (52%) showed only VMT, whereas 35/73 eyes (48%) also showed coexisting alterations at baseline. VMT resolved in 40/73 eyes (55% of cases). Our model disclosed VMT reflectivity as the most involved parameter in VMT resolution. VMT size showed less influence on the success of ocriplasmin treatment. ERM was negatively associated with VMT resolution. Moreover, VMT reflectivity values and ERM represented the most important parameters for the onset of vitreomacular complications.

## Introduction

Vitreomacular traction (VMT) constitutes the evolution of a persistent vitreomacular adhesion (VMA) leading to several eyesight disturbances, including distortion, metamorphopsia and visual acuity reduction^1^. Current treatments include ocriplasmin intravitreal injection or pars plana vitrectomy. Ocriplasmin is a proteolytic enzyme that breaks down the fibronectin and laminin components of the pathological vitreoretinal interface, causing a lysis of the VMT^2^. The National Institute for Health and Care Excellence (NICE) guidelines for the use of intravitreal ocriplasmin pinpoint VMT patients with no evidence of an epiretinal membrane (ERM), with a stage II full-thickness macular hole (FTMH) less than 400 µm in diameter and/or severe symptoms, underlying the importance of patient selection in achieving successful treatment^1,3^. However, the VMT response to ocriplasmin treatment is highly heterogeneous and there is no consensus regarding the effectiveness of this therapy.

In the present study we adopted an artificial intelligence-based mathematical model based on structural optical coherence tomography (OCT) quantitative parameters to predict VMT resolution secondary to ocriplasmin intravitreal injection.

## Methods

The study was designed as a retrospective case series. Consecutive patients affected by focal VMT, with a minimum follow-up of 1 year, were recruited at the Ophthalmology Unit of San Raffaele Hospital, Milan, Italy. All the participants involved in this study provided informed consent. The study was approved by the Ethical Committee of the Vita-Salute San Raffaele University in Milan and conducted in accordance with the Declaration of Helsinki. Exclusion criteria were: high media opacities, any other ophthalmological disorder, ophthalmologic surgery within the last six months, any systemic condition potentially affecting the analyses. Ophthalmologic examination included best corrected visual acuity (BCVA) measurement using standard ETDRS charts, slit lamp biomicroscopy of anterior and posterior segments and Goldmann applanation tonometry. Structural OCT acquisitions (Spectralis HRA + OCT; Heidelberg Engineering, Heidelberg, Germany) included raster, radial and dense scans with a high number of frames (ART > 25) and enhanced depth imaging (EDI). We measured central macular thickness (CMT) and the maximal linear dimension (VMT size) before and after treatment, and a blinded researcher quantified baseline VMT reflectivity with ImageJ software before the treatment^4^. We used structural OCT images to identify coexisting alterations and the development of further vitreomacular alterations over the whole follow-up. We considered the following alterations: epiretinal membrane (ERM), FTMH, lamellar MH, pseudohole.

In order to perform a longitudinal analysis of BCVA and CMT by means of two repeated measures ANOVAs, patients were stratified as follows: VMT without coexisting baseline alterations with VMT resolution (pure_detach), VMT with coexisting baseline alterations and VMT resolution (comorb_detach), VMT without coexisting baseline alterations without VMT resolution (pure_no_detach), VMT with coexisting alterations without VMT resolution (comorb_no_detach). These four groups were considered as between-subjects factors. We added time as the single within-subjects factor: Pre-Treatment (T0), 1-month Post-Treatment (T1), last follow-up (T2). Age and VTM reflectivity were used as numeric covariates. We applied Pillai’s trace test as a multivariate analysis to account for any potential violations of standard RM-ANOVA.

Prediction of complications at T2, regarded as the development of vitreomacular alterations or anatomical worsening of a pre-existing condition, was performed by means of a logistic model. The following parameters were included: BCVA, CMT, VMT Reflectivity, and ERM at baseline.

To obtain a robust measure of prediction accuracy, the following pipeline was adopted: a random selection of 70% of the total sample (the training set) was chosen to train the logistic model. After estimation, the model was tested on the remaining 30% of the sample (the test sets). Both overall prediction accuracy and the significance of the parameters included in the model were retained. The procedure was run 1000 times, using different samples each time, for both the training and test sets. At each iteration, these parameters were computed and stored for subsequent analysis. It was decided to run the model 1000 times to prevent the conclusion being potentially biased by outliers. Furthermore, the sampling was guided in order to guarantee the same between train and test sets for each of our groups. However, it is worth pointing out that grouping was not included in the model itself, so that only quantitative a priori objective measures were entered, thus facilitating generalization for future purposes.

We adopted a similar approach to try to predict treatment success based on the following parameters measured at baseline.

All statistical analyses and data visualization were performed by means of R language, release 3.6.0 under R studio software^5,6^. The statistical threshold was set at 0.05; we adopted Bonferroni correction for post-hoc tests to provide conservative results.

## Results

### Overall findings

We recruited 80 patients affected by VMT. Seven patients were excluded because of media opacities. Seventy-three eyes of 73 patients (30 males; mean age 73 ± 9) were included in the analysis. The mean follow-up duration was 2.6 ± 1.1 years (range 1–4 years). Mean baseline BCVA was 0.38 ± 0.18 LogMAR, improving to 0.26 ± 0.20 at the end of the follow-up (*p* < 0.01). Baseline CMT was 431 ± 118 µm, improving to 393 ± 122 µm at the end of the follow-up (*p* < 0.01). Among the whole cohort, 38/73 eyes (52%) showed only VMT, whereas 35/73 eyes (48%) also showed coexisting alterations at baseline. In particular, 18 eyes showed the presence of a ERM, whereas 17 patients revealed a MH. Before treatment, 46/73 eyes (51%) were pseudophakic. VMT resolved in 40/73 eyes (55% of cases; 22/40 eyes pseudophakic).

### Separate analysis of patients with and without VMT resolution

In order to perform separate analyses, patients were stratified as follows: VMT without coexisting baseline alterations with VMT resolution (pure_detach) (20 eyes), VMT with coexisting baseline alterations and VMT resolution (comorb_detach) (20 eyes), VMT without coexisting baseline alterations without VMT resolution (pure_no_detach) (18 eyes), VMT with coexisting alterations without VMT resolution (comorb_no_detach) (15 eyes). No age difference was found between all the subgroups (*p* > 0.05). A worsening of co-existing alterations at baseline or the development of further vitreomacular interface complications were observed in 3/20 eyes (15%) in the pure_detach group, 15/20 eyes (75%) in the comorb_detach group, 8/18 eyes (44%) in the pure_no_detach group and 15/15 eyes (100%) in the comorb_no_detach group. These alterations were found to break down as follows: ERM in 30/41 eyes (73%), FTMH in 10/41 (24%), lamellar MH in 14/41 eyes (34%) and pseudohole in 4/41 eyes (10%). Remarkably, the 3 eyes in the pure_detach group developed only ERM.

### Subgroup analysis of BCVA

Pure_detach patients showed significantly better baseline BCVA than comorb_detach, pure_no_detach and comorb_no_detach eyes (*p* < 0.01), and maintained the best BCVA values even after treatment and at the end of the follow-up (*p* < 0.01). All the other subgroups displayed similar baseline BCVA values (*p* > 0.05). Comorb_no_detach eyes showed the worst BCVA values at the end of the follow-up, compared with the other three subgroups (*p* < 0.01).

One month after treatment, only pure_detach eyes showed significant BCVA improvement, compared with baseline values (*p* < 0.01). A further BCVA improvement was also reached at the end of the follow-up (*p* < 0.01). Comorb_detach and pure_no_detach eyes displayed significant BCVA improvements at the end of the follow-up, compared with their baseline values (*p* < 0.01), whereas comorb_no_ detach eyes recorded the worst BCVA values, with a slightly significant worsening over the follow-up (*p* < 0.05). All the values are reported in Table 1.

**Table 1:** Subgroup analysis in vitreomacular traction.

Subgroup analysis in vitreomacular traction						
Group	Pure_detach	Comorb_detach	Pure_no_detach	Comorb_no_detach		
Group number	1	2	3	4		
N. of patients	20	20	18	15		
**Mean** ± **STD values**						
F/U Duration (years)	2.5 ± 1.1	2.3 ± 0.8	2.5 ± 0.9	2.6 ± 1.2		
Age	72 ± 13	70 ± 5	76 ± 8	73 ± 8		
LogMAR BCVA baseline	0.28 ± 0.12	0.46 ± 0.21	0.36 ± 0.20	0.42 ± 0.12		
LogMAR BCVA 1-month	0.23 ± 0.10	0.42 ± 0.20	0.34 ± 0.16	0.45 ± 0.15		
LogMAR BCVA last F/U	0.08 ± 0.15	0.29 ± 0.21	0.27 ± 0.10	0.45 ± 0.11		
CMT baseline (µm)	391 ± 89	431 ± 67	472 ± 139	437 ± 165		
CMT 1-month (µm)	359 ± 56	394 ± 65	469 ± 134	440 ± 167		
CMT last F/U (µm)	327 ± 39	356 ± 71	465 ± 130	443 ± 171		
VMT size baseline (µm)	428 ± 143	365 ± 157	532 ± 113	393 ± 201		
VMT size last F/U (µm)	0 ± 0	0 ± 0	515 ± 135	378 ± 215		
VMT reflectivity baseline	110 ± 12	124 ± 15	147 ± 21	154 ± 26		
Complications	3/20 (15%)	15/20 (75%)	8/18 (44%)	15/15 (100%)		
**p Values baseline vs 1-month**						
Group number	1	2	3	4		
LogMAR BCVA	0.007*	0.082	0.113	0.04*		
CMT (µm)	0.002*	< 0.001*	0.153	0.560		
**p Values baseline vs last follow-up**						
Group number	1	2	3	4		
LogMAR BCVA	< 0.001*	< 0.001*	0.005*	0.04*		
CMT (µm)	< 0.001*	< 0.002*	0.15	0.56		
**p Values 1-month vs last follow-up**						
Group number	1	2	3	4		
LogMAR BCVA	< 0.001*	< 0.001*	0.002*	0.87		
CMT (µm)	< 0.001*	< 0.001*	0.15	0.56		
**p Values between groups**						
Parameter	1 vs 2	1 vs 3	1 vs 4	2 vs 3	2 vs 4	3 vs 4
F/U Duration (years)	> 0.05	> 0.05	> 0.05	> 0.05	> 0.05	> 0.05
Age	> 0.05	> 0.05	> 0.05	> 0.05	> 0.05	> 0.05
LogMAR BCVA baseline	0.002*	0.02*	0.001*	0.15	0.54	0.32
LogMAR BCVA 1-month	0.0008*	0.02*	< 0.001*	0.17	0.65	0.04*
LogMAR BCVA last F/U	0.001*	< 0.001*	< 0.001*	0.77	0.009*	< 0.001*
CMT baseline (µm)	> 0.05	> 0.05	> 0.05	> 0.05	> 0.05	> 0.05
CMT 1-month (µm)	0.03*	0.001*	0.04*	0.03*	0.26	0.59
CMT last F/U (µm)	0.13	< 0.001*	0.006*	0.003*	0.04*	0.67
VMT size baseline (µm)	0.24	0.01*	0.55	< 0.001*	0.65	0.01*
VMT size last F/U (µm)	< 0.001*	< 0.001*	< 0.001*	< 0.001*	< 0.001*	0.03*
VMT reflectivity baseline	0.002*	< 0.001*	< 0.001*	< 0.001*	< 0.001*	0.38
Complications	< 0.001*	0.003*	< 0.001*	0.002*	0.06	< 0.001*

The following abbreviations are used: best-corrected visual acuity (BCVA), central macular thickness (CMT), vitreomacular traction (VMT). Statistically significant values are marked by asterisks (*).

### Subgroup analysis of CMT

Baseline CMT values were similar between all subgroups (*p* > 0.05). The best CMT values were shown by pure_detach eyes both one month after treatment and at the end of the follow-up (*p* < 0.01). Comorb_detach eyes displayed significantly better CMT values than pure_no_detach and comorb_no_detach eyes, both one month after treatment and at the end of the follow-up (*p* < 0.01), whereas pure_no_detach and comorb_no_detach showed similar CMT values over the entire follow-up (*p* > 0.05). Both pure_detach and comorb_detach revealed CMT improvements 1 month after treatment, compared with their baseline values (*p* < 0.01), and further significantly improved CMT was found at the end of the follow-up (*p* < 0.01). Pure_no_detach and comorb_detach eyes showed no significant CMT changes over the entire follow-up (*p* > 0.05). All the values are reported in Table 1.

### Structural OCT properties of VMT

Pure_no_detach eyes displayed the highest VMT values at baseline, compared with the other subgroups (*p* < 0.01), and maintained higher values than comorb_no_detach eyes even at the end of the follow-up (*p* < 0.01). VMT values of pure_detach, comorb_detach and comorb_no_detach eyes were similar at baseline (*p* > 0.05).

**Figure 1:**
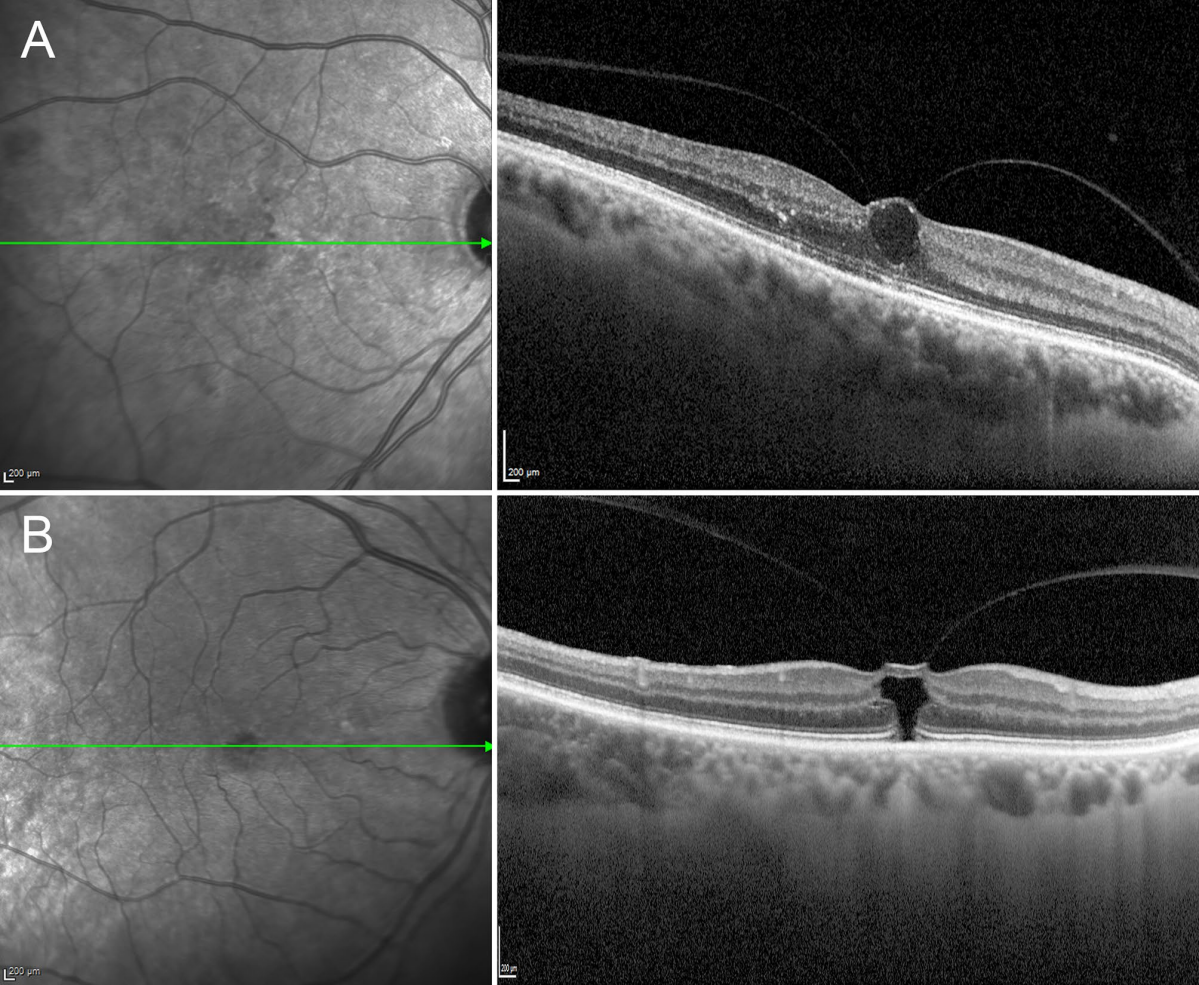
A case of low reflectivity vitreomacular traction and a case of high reflectivity vitreomacular traction.

Pure_no_detach and comorb_no_detach eyes showed similar VMT reflectivity values (*p* > 0.05); these proved significantly higher than pure_detach and pure_no_detach eyes (*p* < 0.01). Moreover, the lowest VMT reflectivity values were shown by pure_detach eyes (*p* < 0.01) (Fig. 1). Complete data are reported in Table 1.

### Treatment success

Mean overall accuracy was only 52% (SEM = 0.007%). Among the parameters included, only VMT reflectivity was found to be significantly involved in predicting treatment success (994 out of 1000 cases). Detailed data are listed in Table 2.

**Table 2:** Prediction of ocriplasmin treatment success.

Parameter	LogMAR BCVA Baseline		CMT Baseline		VMT size baseline		VMT Reflectivity		ERM Baseline	
Measure	Mean	SEM	Mean	SEM	Mean	SEM	Mean	SEM	Mean	SEM
Estimate	5.74	0.44	− 0.007	0.0004	− 0.006	0.0002	− 0.18	0.009	0.29	0.064
Z score	1.27	0.046	− 1.29	0.04	− 1.60	0.052	− 3.06	0.023	0.25	0.045
N. of times	75/1000		8/1000		252/1000		994/1000		0/1000	

Overall results gathered from the 1000 Logistic Models run by randomly assigning subjects to training and test sets (70%/30%). Average results are reported for estimate and z score, in addition to the number of times each parameter proved significantly involved in predicting complications at the last follow-up.

**Figure 2:**
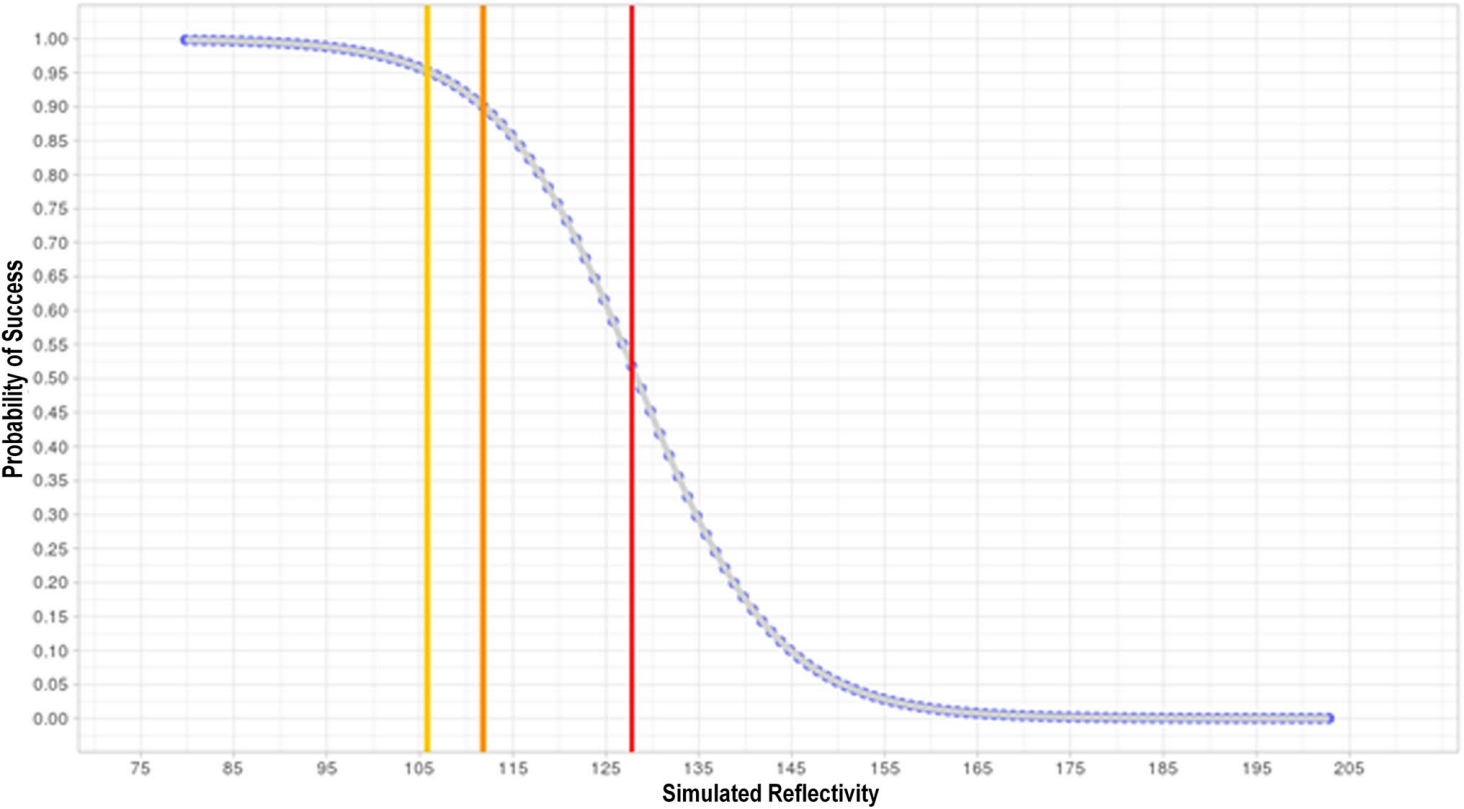
Simulation of dependence of probability of success of reflectivity. Colored lines indicate safety threshold. Gold line, at reflectivity level 105.8 corresponds to 95% success threshold; orange line, at reflectivity level 111.8 corresponds to 90% success threshold, while the red line, at reflectivity level 127.8, corresponds to 50% success threshold.

To further show VMT reflectivity importance, we simulated with a varying range of VMT reflectivity while keeping constant the other parameters to sample means of our patients; our goal was to show a potential probability risk based on the VMT reflectivity measured before treatment. As Fig. 2 makes plain, the lower the VMT reflectivity, the higher the chance of treatment success. For visualization purposes, we highlighted three values: 105.8, corresponding to 95% success threshold, 111.8, corresponding to 90% success threshold, and 127.8, corresponding to 50% success threshold. Less influence was observed concerning VMT size (252 out of 1000 cases).

### Prediction of vitreomacular complications

The Logistic Model’s mean overall accuracy prediction was 88.13% (sem = 0.0065%). VMT reflectivity proved to be significantly involved in predicting the onset of vitreomacular complications or the worsening of a pre-existing condition at the end of the follow-up (793 out of 1000 times), followed by ERM presence at baseline (771 out of 1000 times), and baseline BCVA (664 out of 1000). The influence of VMT size and CMT baseline values was less marked. Detailed statistics obtained from the 1000 times the Logistic Model was run are shown in Table 3.

**Table 3:** Prediction of the onset of complications up to the end of the follow-up.

Parameter	LogMAR BCVA Baseline		CMT Baseline		VMT size Baseline		VMT Reflectivity		ERM Baseline	
Measure	Mean	SEM	Mean	SEM	Mean	SEM	Mean	SEM	Mean	SEM
Estimate	7.34	0.30	0.001	0.0006	− 0.004	0.0005	0.059	0.002	13.42	1.62
Z score	2.08	0.042	0.12	0.08	− 1.34	0.047	2.206	0.038	2.28	0.13
N. of times	664/1000		14/1000		143/1000		793/1000		771/1000	

Overall results gathered from the 1000 Logistic Models run by randomly assigning subjects to training and test sets (70%/30%). Average results are reported for estimate and z score, in addition to the number of times each parameter proved significantly involved in predicting complications at the end of the follow-up.

## Discussion

Although current indications are that ocriplasmin is an effective treatment for VMT, its success rate is still variable^1,3,7^.

In the present study, we analyze a predictive model for assessing the success of ocriplasmin intravitreal injection in resolving VMT. We observed complete VMT resolution in 55% of our case series. These data agree with previous investigations assessing the percentages of patients showing VMT resolution after ocriplasmin treatment^8,9^.

A separate analysis of VMT patients, stratified according to the presence of co-existing alterations at baseline, revealed that the resolution of the VMT is associated with BCVA and CMT improvements, with a negative influence exerted by the presence of ERM or MH at baseline. Patients with only VMT at baseline and with no VMT resolution also showed BCVA improvement at the end of the follow-up. This could be explained as a kind of stabilization of the vitreomacular condition, in the absence of the onset of further alterations.

Study of the VMT’s structural OCT properties revealed that VMT reflectivity was highest in patients not showing VMT resolution after treatment. Moreover, among patients showing VMT resolution, VMT reflectivity proved to be significantly higher in patients with co-existing alterations at baseline. VMT reflectivity was the most important factor in our model in predicting ocriplasmin treatment success. Specifically, we identified three values: 105.8 (95% success), 111.8 (90% success) and 127.8 (50% success). Furthermore, although VMT size proved similar between subgroups, it was found to play a part in treatment success, although less than VMT reflectivity. This finding agreed with previous studies showing an influence of the size of the adhesion on the treatment success^2^.

VMT reflectivity also proved to be a powerful predictor of the onset of complications and the worsening of co-existing alterations. The presence of ERM at baseline and the starting BCVA values also exerted a strong influence. In contrast, VMT size and CMT baseline values were found to have a weaker influence in this kind of analysis.

The vitreous and vitreoretinal interfaces are made up of a precise set of molecules, including collagen, fibronectin and laminin. These components are produced in a highly dynamic manner and may vary according to age, inducing morphological changes of the vitreoretinal interface^10,11^. The onset of anatomical modifications, such as partial posterior vitreous detachment and VMT may trigger splits of the inner limiting membrane, allowing glial cells, astrocytes and myofibroblasts to migrate and proliferate, with several components, including epiretinal fibroglial membranes and collagen, being deposited between the retinal and vitreous surfaces; another result is the onset of ERM^12–16^. Further worsening of the molecular composition of the vitreoretinal interface and the increased tractional force may cause the development of further complications, such as MH^1^. Ocriplasmin fits into this highly complex context by degrading fibronectin and laminin and by inducing ultrastructural modifications of the vitreoretinal interface^17,18^. However, the almost 50% treatment success suggests that VMT composition may vary between patients, thus influencing the vitreolytic effect of ocriplasmin.

Our findings suggest that VMT reflectivity may be the expression of the molecular composition of VMT. Higher reflectivity values might be related to the VMT’s more organized composition, which in turn is associated with both the success rate of ocriplasmin-related vitrolysis and the worsening of the vitreomacular interface over the follow-up.

We are aware that our study has several limitations, particularly in relation to its retrospective design, and the number of patients is too small to draw definitive conclusions. Also, the mathematical model applied needs to be validated by a larger controlled cohort of patients. Moreover, structural OCT is known to be potentially influenced by image artifacts.

Nevertheless, the present study should be viewed as a pilot project that attempts to provide new biomarkers to help decide whether to opt for ocriplasmin therapy in common clinical practice. Further prospective studies are warranted to examine the true effectiveness of structural OCT biomarkers in the management of VMT by means of ocriplasmin.
